# Supplementary figures and images for: Expanding the Diversity of Myoviridae Phages Infecting Lactobacillus plantarum—A Novel Lineage of Lactobacillus Phages Comprising Five New Members
Source: Viruses. 2019 Jul 4;11(7):611. doi: 10.3390/v11070611 (PMC6669764; doi:10.3390/v11070611)

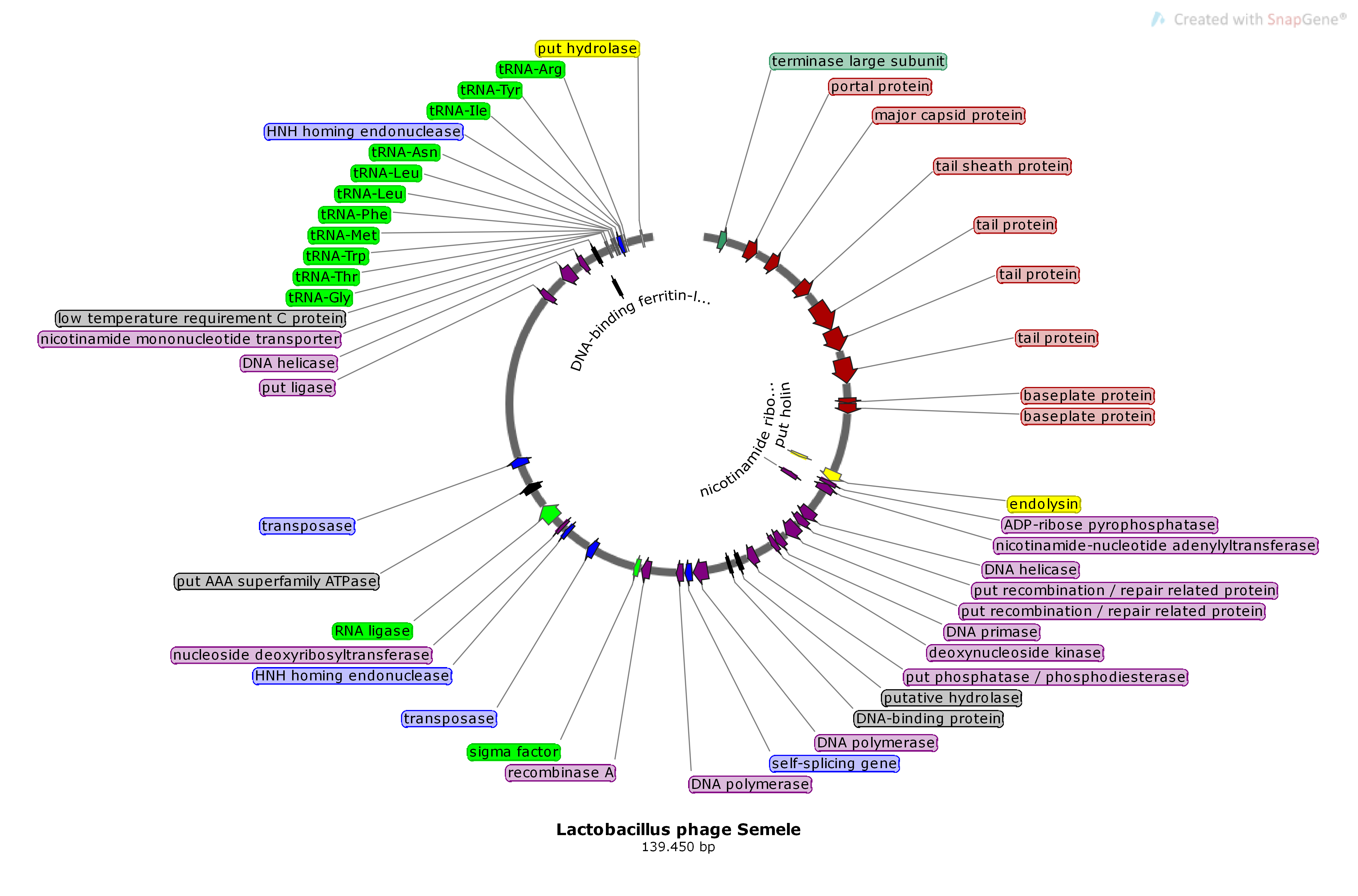

Supplement: Supplementary file 1 [file viruses-11-00611-s001.zip › Figure S1.tiff]

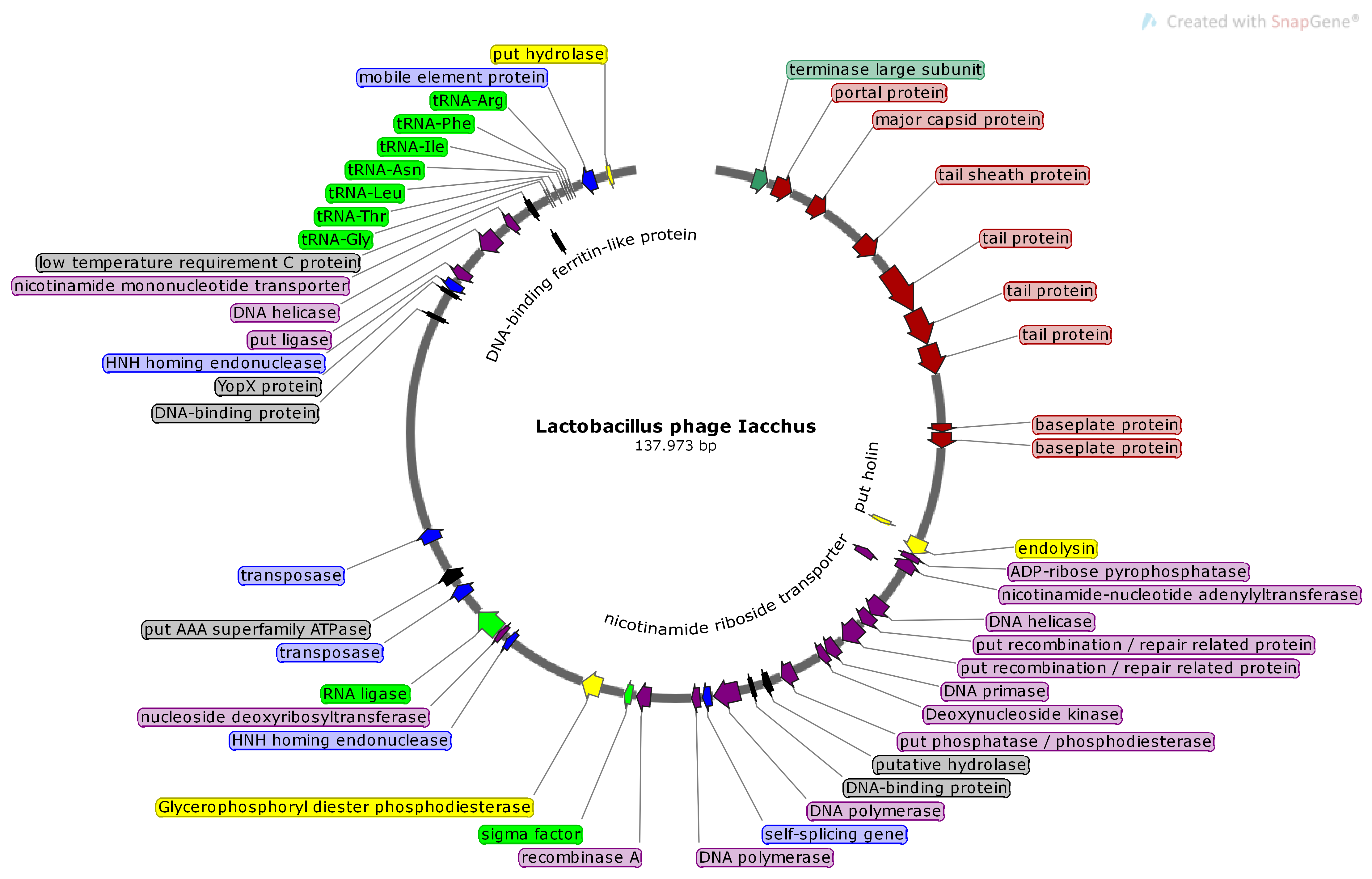

Supplement: Supplementary file 1 [file viruses-11-00611-s001.zip › Figure S2.tiff]

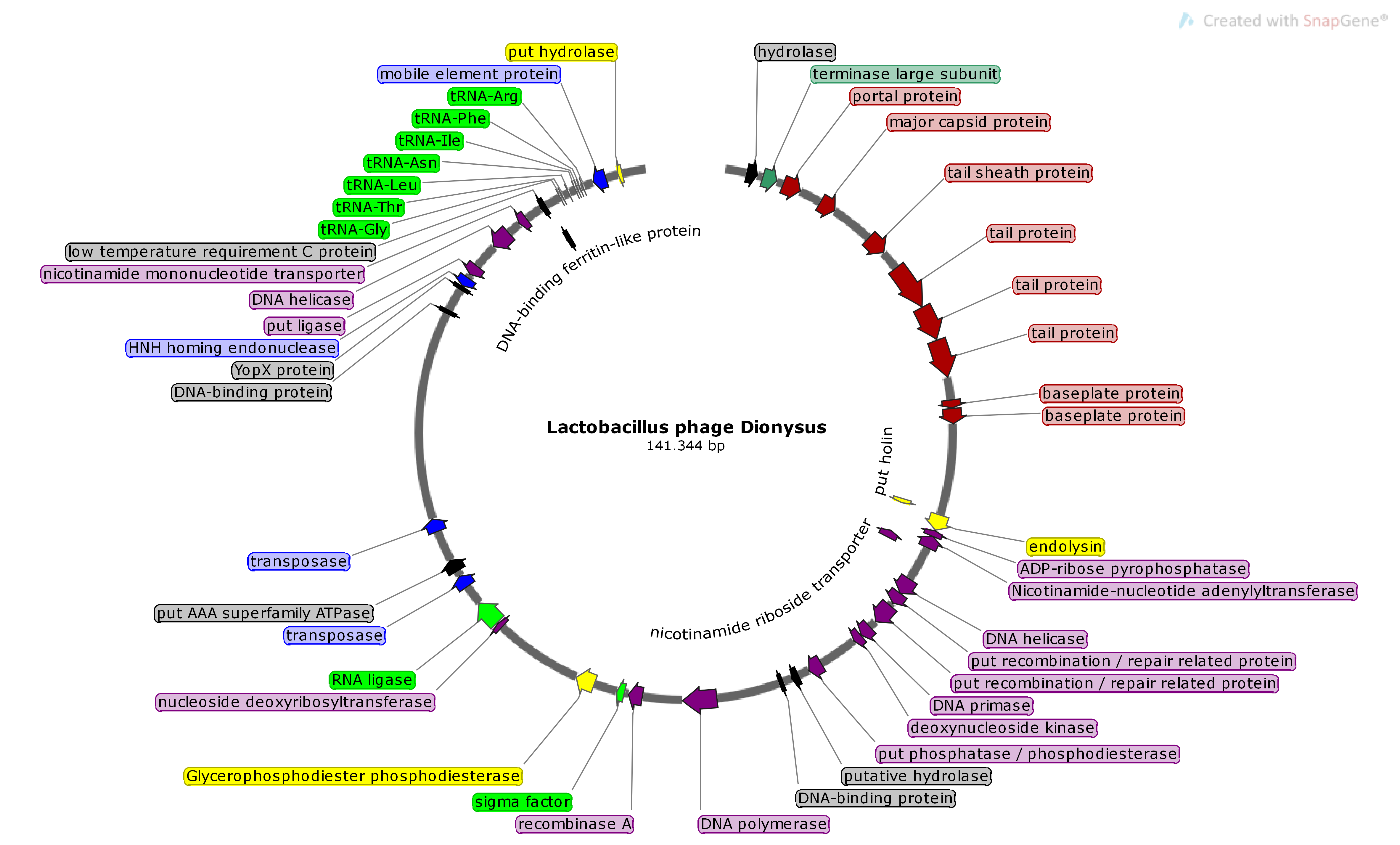

Supplement: Supplementary file 1 [file viruses-11-00611-s001.zip › Figure S3.tiff]

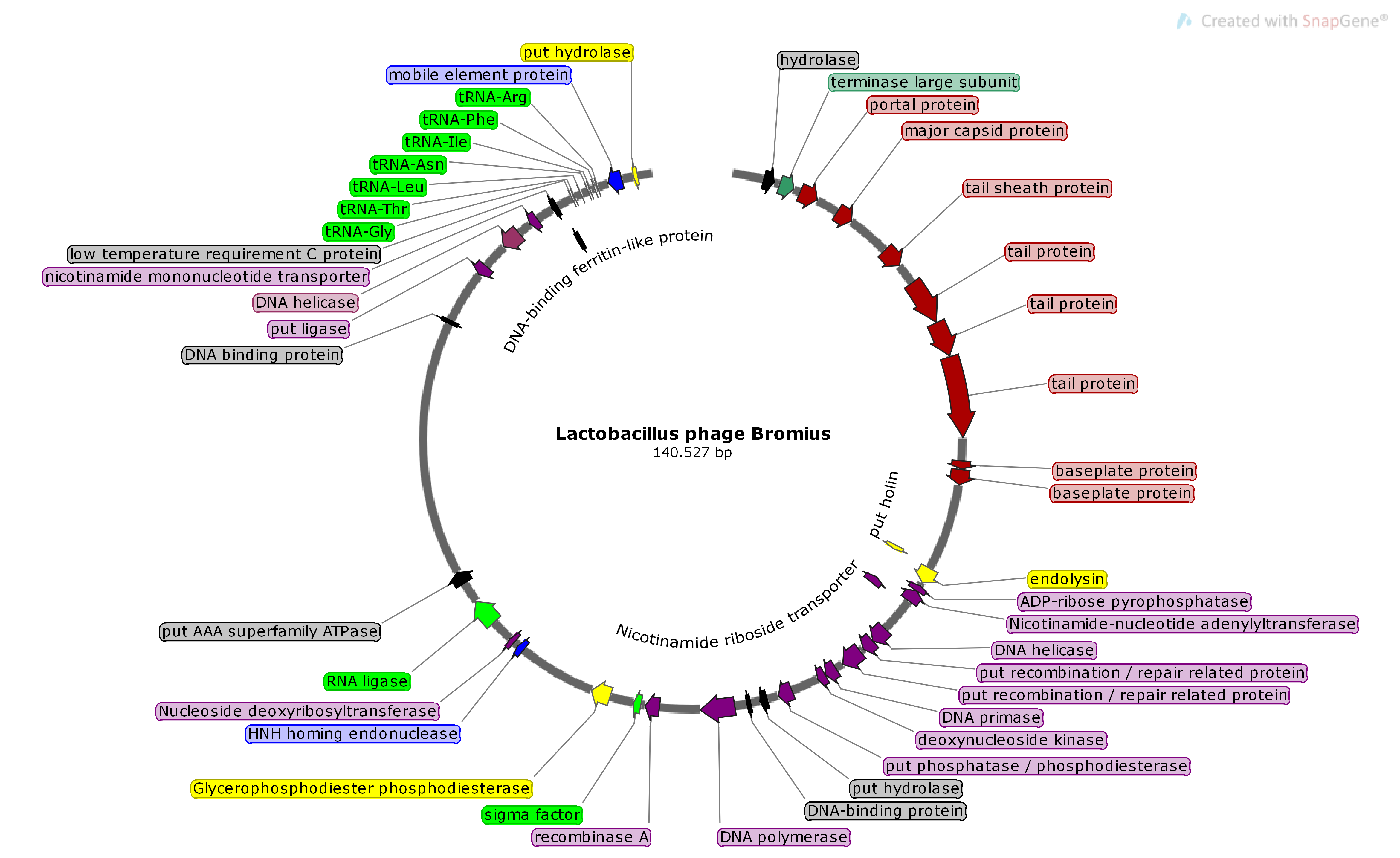

Supplement: Supplementary file 1 [file viruses-11-00611-s001.zip › Figure S4.tiff]

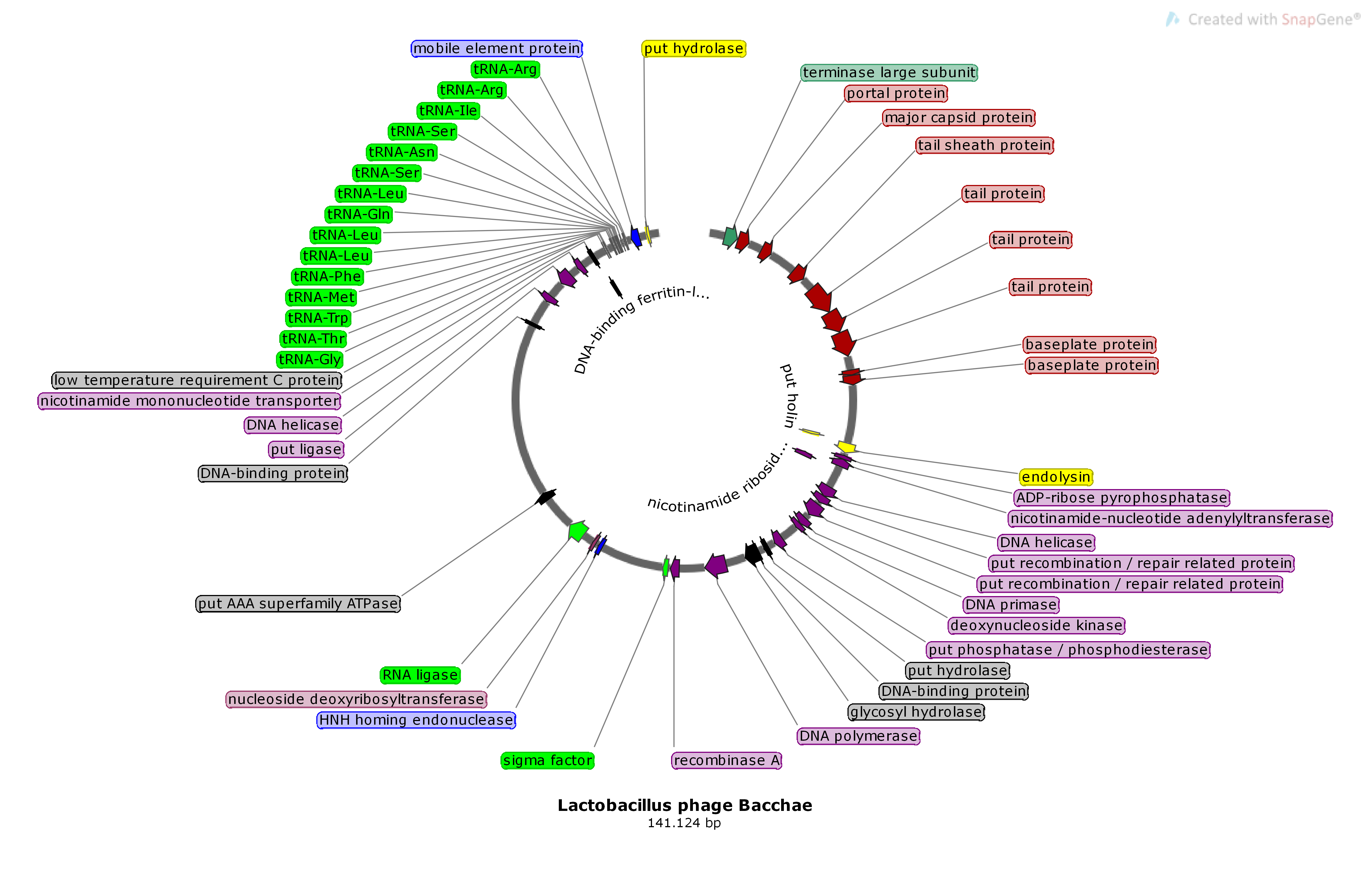

Supplement: Supplementary file 1 [file viruses-11-00611-s001.zip › Figure S5.tiff]

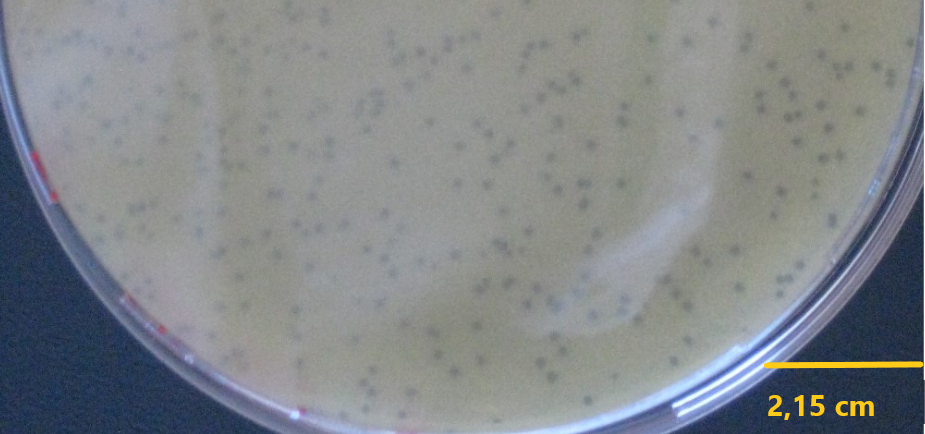

Supplement: Supplementary file 1 [file viruses-11-00611-s001.zip › Figure S6.tiff]
